# Quantum annealing and computation: challenges and perspectives

**DOI:** 10.1098/rsta.2021.0419

**Published:** 2023-01-23

**Authors:** Bikas K. Chakrabarti, Hajo Leschke, Purusattam Ray, Tatsuhiko Shirai, Shu Tanaka

**Affiliations:** ^1^ Saha Institute of Nuclear Physics, Kolkata 700064, India; ^2^ Indian Statistical Institute, Kolkata 700108, India; ^3^ Institut für Theoretische Physik, Universität Erlangen-Nürnberg, 91058 Erlangen, Germany; ^4^ The Institute of Mathematical Sciences, Taramani, Chennai 600113, India; ^5^ Department of Computer Science and Communications Engineering, Waseda University, Tokyo 169-8555, Japan; ^6^ Department of Applied Physics and Physico-Informatics, Keio University, Yokohama 223-8522, Japan

**Keywords:** NP-hard problems, quantum Ising spin glass, quantum computation, quantum tunnelling, quantum annealing

## Abstract

In the introductory article of this theme issue, we provide an overview of quantum annealing and computation with a very brief summary of the individual contributions to this issue made by experts as well as a few young researchers. We hope the readers will get the touch of the excitement as well as the perspectives in this unusually active field and important developments there.

This article is part of the theme issue ‘Quantum annealing and computation: challenges and perspectives’.

## 1. Introduction

The development and success of digital computers eclipsed the parallel development of analogue computation. However, in recent years, it is realized that in many complex problems, instead of searching sequentially (using digital algorithms) for the maximum depth of a rugged landscape, one can search (in an ‘analogue’ way of physical annealing search for the crystal from the melt) using the idea and physics of metallurgical annealing. In the seminal paper entitled ‘Optimization by simulated annealing’ [1], Kirkpatrick, Gelatt and Vecchi proposed this novel technique. Though it turns out to be remarkably successful for finding practical solutions to many optimization problems, it is not so for problems like the N-city Travelling Salesman Problem or the problem of finding the ground state(s) of a spin-glass model with N (Ising) spins being ‘randomly frustrated’ due to the random interactions between them. These examples belong to the NP-hard computational problems, because the search time for finding practical solutions grows faster with N than any polynomial in N.

In the second example the bottleneck was identified. Extensive study (e.g. [2,3]) of the dynamics of frustrated random systems like the Ising spin glasses, in particular of the Sherrington–Kirkpatrick (SK) variety [4], showed that its (free) energy landscape (in the spin-glass phase) is extremely rugged and the barriers, separating the local valleys, often become of order N, inducing non-ergodicity or localization. Classical (thermal) fluctuations like those in the simulated annealing technique fail to help the system get out of such deep valleys (at random locations or configurations, due to random frustration) as the escape probability at temperature $T_{c}$ (classical fluctuations) is of order $\exp(-N/T_{c})$ and the annealing time cannot be bounded by any polynomial in N.

Following the initial study [5] of phase transition of transverse (quantum) Ising spin glasses, the idea proposed by Ray *et al*. [6] was that quantum fluctuations in the transverse SK model can perhaps lead to some escape routes to ergodicity or quantum fluctuation-induced delocalization (at least in the low-temperature region of the spin-glass phase) by allowing tunnelling through such macroscopically tall but thin barriers which are difficult to scale using classical fluctuations. This is based on the observation that the escape probability due to quantum tunnelling, from a valley with single barrier of height N and width w, scales as $\exp(-\sqrt{N}w/\Gamma_{q})$, where $\Gamma_{q}$ represents the quantum fluctuation strength (or tunnelling probability) [7]. This extra handle through the barrier width w (absent in thermal escape probability) can help in its vanishing limit. This has led to some important clues (see e.g. the discussions in this regard in [8]). In fact, the recent rigorous study [9] on the transverse SK spin glass provides some support for this aspect in the low-temperature limit, implying the possibility of quantum annealing (see also [10]). In parallel the adiabatic quantum computation in the same context of such NP-hard computational problems was developed [11].

The quantum annealing technique was finally launched through the paper by Kadowaki & Nishimori [12] and its experimental demonstration by Brooke *et al.* [13]. Soon, a superconducting-circuit quantum Ising glass annealing machine was developed [14] (and later marketed) by the D-wave systems. Since then, a revolution has taken place through a surge of outstanding papers both in theory and experiment and these intense and massive studies in the last two decades led finally to the birth of this new age of quantum information and technologies.

Several reviews on quantum annealing (e.g. [15,16]), on adiabatic quantum computation (e.g. [17,18]) and books (e.g. [19]) have already been published. Still, in view of the unusually fast growth of the field we planned to have a compendium connecting the key results over the last few years and include also some reviews on these and our present understanding of them, by active scientists and experts. The prime objective of this issue is to bridge the gap between what was known before (say 5 years ago) and what seem to be the main challenges in recent times. The present timing of such an issue is more than appropriate as there has been a tremendous growth of studies in these fields over the past few years. We now introduce very briefly the contents of this theme issue in four different sections.

## 2. Quantum spin-glass and annealing

The paper entitled ‘Quantum annealing: an overview’ [20] by A. Rajak, S. Suzuki, A. Dutta and B. K. Chakrabarti reviews the theoretical physics background of quantum annealing. The results of traditional as well as recent papers on quantum annealing are systematically discussed and presented. The paper entitled ‘Greedy parameter optimization for adiabatic quantum annealing’ [21], by Kadowaki & Nishimori, proposes an interesting use of the fidelity as a cost function, assuming a knowledge of the target state, for the annealing dynamics. In the paper entitled ‘Threshold theorem in isolated quantum dynamics with stochastic control errors’ [22], Okuyama *et al.* consider the Schrödinger equation for a system with a Hamiltonian smoothly depending on time, but being perturbed by operators multiplied by Gaussian white noise. The noise term models the effects of stochastic control errors in the noiseless system during the (computational) time T>0. The authors prove that a given eigenstate of a time-independent observable can be obtained at time T with high probability by sufficiently many measurements, if the noise strength is smaller than 1/T. This is of interest for quantum annealing and adiabatic computation. In the paper entitled ‘Role of quantum fluctuations in inducing ergodicity in the spin glass phase and its effect in quantum annealing’ [23], Mukherjee reviews numerical studies of the (quantum) SK model with a transverse field performed by him in collaboration with Chakrabarti & Rajak. The central message is that the spin glass phase subdivides into two regions, one being dominated by quantum critical behaviour (with ergodic features) and the other one by classical critical behaviour (with non-ergodic features). While some aspects of the phase diagram may suffer from finite-size effects, it provides a coherent picture which future researchers should keep in mind when studying the model by whatever method. The paper entitled ‘Statistics of the number of defects after quantum annealing in a thermal environment’ [24] by Suzuki *et al.* studies the statistics of the kink number generated by quantum annealing in a one-dimensional transverse Ising model coupled to a bosonic heat bath. The theoretical result is confirmed by numerical simulation. The simulation using D-Wave’s quantum annealer is also discussed.

## 3. Adiabatic quantum computation

In the paper entitled ‘Quantum adiabatic theorem for unbounded Hamiltonians with a cutoff and its application to superconducting circuits’ [25] by Lidar & Mozgunov, the authors introduce a new quantum adiabatic theorem that provides rigorous bounds on the adiabatic time scale for a variety of systems, including originally unbounded Hamiltonians by applying a cut-off.

## 4. Spin dynamics, topological and optical properties etc. in quantum annealers and computers

The paper entitled ‘Trajectory phase transitions in non-interacting systems: all-to-all dynamics and the random energy model’ [26] by Garrahan *et al.* addresses the problem of minimization via trajectory sampling of random cost functions like the energy function in a simple mean-field spin glass model like a random energy model. This has direct implications in training neural networks. The paper entitled ‘Optimization with photonic wave based annealers’ [27] by Prabhakar *et al.* develops two different types of photonic Ising machines and compares the performances with a state-of-the-art classical solver and a quantum annealer on two combinatorial optimization problems. The paper entitled ‘Simulations of frustrated Ising Hamiltonians using quantum approximate optimization’ [28] by Lotshaw *et al.* investigates the properties of the ground state of frustrated magnetic materials using QAOA, a quantum computer-based optimization algorithm. In the paper by Dridi *et al*. entitled ‘Understanding domain-wall encoding theoretically and experimentally’ [29] the authors consider domain-wall qubit encodings using integer valued variables and map the original optimization problem into a QUBO of binary variables.

## 5. Network structure and growth statistics for quantum computation and information

The paper entitled ‘Development of research network on quantum annealing, computation and information using Google Scholar data’ [30] by Sinha analyses the time evolution of research networks by extracting data on papers related to quantum annealing and quantum computation using Google Scholar.

## 6. Concluding remarks

The development of quantum annealing and adiabatic quantum computers has been unique in physics of many-body quantum systems as well as in quantum engineering of information processing and has become a very active field of research (see e.g. [30] in this issue, showing that the research publications in this field are growing every 5 years by a factor e (Euler number ≃2.7), compared to some other very active contemporary research fields taking almost double that time to grow by the same factor). We hope the issue will provide useful study materials and will be sufficiently informative to the interested researchers, younger ones in particular.

## Data Availability

This article has no additional data.
